# Detection of COVID-19 in X-ray Images Using Densely Connected Squeeze Convolutional Neural Network (DCSCNN): Focusing on Interpretability and Explainability of the Black Box Model

**DOI:** 10.3390/s22249983

**Published:** 2022-12-18

**Authors:** Sikandar Ali, Ali Hussain, Subrata Bhattacharjee, Ali Athar, Abdullah, Hee-Cheol Kim

**Affiliations:** 1 Department of Digital Anti-Aging Healthcare, Inje University, Gimhae 50834, Republic of Korea; 2 Department of Computer Engineering, Inje University, Gimhae 50834, Republic of Korea; 3 Institute of Digital Anti-Aging Healthcare, College of AI Convergence, u-AHRC, Inje University, Gimhae 50834, Republic of Korea

**Keywords:** artificial intelligence, X-ray, COVID-19, classification, detection, AI explainability

## Abstract

The novel coronavirus (COVID-19), which emerged as a pandemic, has engulfed so many lives and affected millions of people across the world since December 2019. Although this disease is under control nowadays, yet it is still affecting people in many countries. The traditional way of diagnosis is time taking, less efficient, and has a low rate of detection of this disease. Therefore, there is a need for an automatic system that expedites the diagnosis process while retaining its performance and accuracy. Artificial intelligence (AI) technologies such as machine learning (ML) and deep learning (DL) potentially provide powerful solutions to address this problem. In this study, a state-of-the-art CNN model densely connected squeeze convolutional neural network (DCSCNN) has been developed for the classification of X-ray images of COVID-19, pneumonia, normal, and lung opacity patients. Data were collected from different sources. We applied different preprocessing techniques to enhance the quality of images so that our model could learn accurately and give optimal performance. Moreover, the attention regions and decisions of the AI model were visualized using the Grad-CAM and LIME methods. The DCSCNN combines the strength of the Dense and Squeeze networks. In our experiment, seven kinds of classification have been performed, in which six are binary classifications (COVID vs. normal, COVID vs. lung opacity, lung opacity vs. normal, COVID vs. pneumonia, pneumonia vs. lung opacity, pneumonia vs. normal) and one is multiclass classification (COVID vs. pneumonia vs. lung opacity vs. normal). The main contributions of this paper are as follows. First, the development of the DCSNN model which is capable of performing binary classification as well as multiclass classification with excellent classification accuracy. Second, to ensure trust, transparency, and explainability of the model, we applied two popular Explainable AI techniques (XAI). i.e., Grad-CAM and LIME. These techniques helped to address the black-box nature of the model while improving the trust, transparency, and explainability of the model. Our proposed DCSCNN model achieved an accuracy of 98.8% for the classification of COVID-19 vs normal, followed by COVID-19 vs. lung opacity: 98.2%, lung opacity vs. normal: 97.2%, COVID-19 vs. pneumonia: 96.4%, pneumonia vs. lung opacity: 95.8%, pneumonia vs. normal: 97.4%, and lastly for multiclass classification of all the four classes i.e., COVID vs. pneumonia vs. lung opacity vs. normal: 94.7%, respectively. The DCSCNN model provides excellent classification performance consequently, helping doctors to diagnose diseases quickly and efficiently.

## 1. Introduction

Coronavirus ORONAVIRUS is a type of single-cell RNA virus that causes respiratory complications and infections in patients [1,2]. It belongs to the Coronaviridae family, and it has crown-like spikes on its external surface, hence called coronavirus [3]. Corona is a Latin word that refers to the crown. The novel coronavirus disease, also known as COVID-19, first originated from Wuhan City of China in December 2019 [4]. Within a short period, it has spread worldwide and has engulfed many lives. Considering its rapid spread, the intensity of mortality, and some other severe disease conditions, it was declared a pandemic by the World Health Organization (WHO) [5]. Phylogenetic analysis of genome sequence has been recommended as a novel type of beta coronavirus [6] and is similar to severe acute respiratory syndrome coronavirus (SARS-CoV). This novel virus was named coronavirus disease (COVID-19) on 11 February 2020. Since coronavirus is a contiguous disease and its rate of spread is faster and more severe than that of any other virus, therefore, many countries impose travel restrictions and lockdowns to stop its rapid spread. However, it continues to affect people across the world intermittently. Recently a new variant of this deleterious disease, B.1.1.529 (omicron), was reported in South Africa on 25 November 2021, by World Health Organization [7,8]. Different vaccines [9] have been introduced to deal with this dreadful virus, but new variants appear intermittently. Recently, the delta variant [10] has been found in patients which is more dangerous and contagious than the previous variants. There are several prominent symptoms associated with this disease. The most common symptoms are mild to moderate breathing problems, cough, cold, fever, pneumonia, dyspnea, headache, and the respiratory system of the patient gets infected [11,12]. Furthermore, loss of taste and smell has also been observed as symptoms of COVID-19 [13].

To date (10 January 2022), 298,915,721 confirmed cases of COVID-19 have been reported, including 5,469,303 deaths. Moreover, in America, the number of confirmed cases is recorded as 108,806,129, Europe 108,040,601, South-East Asia 45,406,693, East Mediterranean 17,277,716, Africa 7,542,653, and western Pacific 11,841,165 according to the WHO [14]. This disastrous disease continues to cause massive deaths worldwide and countries are facing serious health and economic problems. Although many researchers have attempted to identify a viable solution to stop the spread of this contagious disease, however, they are unable to achieve remarkable success. To address this challenging issue, there is a dire need to use the latest technologies, such as artificial intelligence-based systems, which can ensure the rapid diagnosis and prognosis of this deleterious disease and, consequently, can propose pro-active measures for the prevention and treatment of this dis-ease. Traditionally, the diagnosis of COVID-19 is mostly performed by polymerase chain reaction (PR-PCR) [15] and is not very effective in diagnosing the disease. Since it is a manual technique, it is a time-consuming process with a low positivity rate [16]. The low sensitivity of PT-PCR means a low rate of detection and diagnosis of the disease via this process, which is tantamount to risking the lives of patients. As this dis-ease is lethal, we cannot compromise and rely on a diagnostic system with low diagnostic accuracy. Furthermore, PT-PCR test kits are very expensive, and many countries have a shortage of this kit; consequently, it causes a delay in the process of dis-ease prevention and treatment drive [17]. Experts and researchers have also proposed chest CT for the detection of COVID [18,19]. Chest CT and radiography are considered fast and inexpensive methods [20]. Chest CT scans are suggested as an effective way to detect COVID because they contain a lot of pathological information and help diagnose the disease with high accuracy considering all the important information. However, to understand the pathological information, analyze the chest CT, and make a correct diagnosis from those images, there is a need for highly professional radiologists [21]. This may also slow down the detection process of this contagious dis-ease. Furthermore, it may not be very effective as it involves human error factors because expert knowledge and extensive experience are required for the correct diagnosis of X-ray or CT images. Owing to artificial intelligence for empowering and enabling us to develop diagnostic systems that can perform the job of radiologists with optimal performance. This approach also speeds up the diagnosis process and subsequent treatment.

The objective of this study was to stratify the Corona-virus patients while using chest X-ray images. Most of the previous research studies were based on transfer learning approaches; however, we did not use pre-trained models, which are meant for color images. Such pre-trained learning methods may not be suitable for chest X-ray images because of the mismatch in learned features between the natural images, for example, ImageNet, and medical images, and may not be able to provide the desired results for real-time detection of the disease. Therefore, we developed a new method based on a densely connected squeeze convolutional neural network (DCSCNN) for the classification of COVID-19 patients. For the development of any machine learning or deep learning model, the quality of data plays a very important role. Therefore, to preprocess the data and enhance the quality of data, we applied image processing techniques for example bias field correction on the X-ray images. We cropped the images and extracted the region of interest (ROI) from the images and applied bias field correction. This technique helped us to enhance the quality of images. These high-quality X-ray images were used for the training of the DCSCNN model. Furthermore, we applied two Explainable AI techniques to ensure the trust, transparency, and explainability of our proposed model. The use of a deep learning-based model will have a remarkable impact on coping with this global crisis and will reinforce the improvement of outcomes in the fight against this disease. The contributions of this study are summarized as follows:

- Developed DCSCNN model for binary and multi-class classification of COVID-19 and other lung disease conditions.
- Preprocessing of the X-ray images by performing intensity inhomogeneity correction to obtain the optimal performance of the model and correct the image intensity.
- Explainable artificial intelligence (XAI) techniques i.e., Grad-CAM and LIME techniques were utilized to interpret and explain the output of the proposed model.
- The classification was performed on chest X-ray images using supervised learning approaches.
- Seven types of classifications have been performed i.e., COVID vs. normal, COVID vs. lung opacity, lung opacity vs. normal, COVID vs. pneumonia, pneumonia vs. lung opacity, pneumonia vs. normal, and COVID vs. pneumonia vs. lung opacity vs. normal.

The remaining sections of this paper are organized as follows: Section 2 presents related work. The materials and methods are discussed in Section 3. The experimental results and analysis are presented in Section 4. Finally, Section 5 presents the conclusions of this study.

## 2. Related Works

Since the emergence of COVID-19, many researchers have attempted to identify solutions to deal with this disease. Researchers have proposed various approaches and used the latest technologies for the diagnosis, detection, classification, and treatment of this contagious disease [22,23]. Narin et al. [24] developed a deep convolutional-based model for the automatic detection of coronavirus (COVID-19). They used 341 chest X-ray images of COVID-19 patients, 2800 normal chest X-ray images, 2772 bacterial, and 1493 viral pneumonia chest X-ray images to develop this automatic detection system. They used five pre-trained CNN models: RestNet101, RestNet152, RestNet50, Inception V3, and Inception-ResNetV2. They created three binary datasets (Dataset-1, Dataset-2, Dataset-3). Three different binary classifications were implemented for four classes: normal, COVID-19, bacterial pneumonia, and viral pneumonia. They also applied five-fold cross-validation in their experiment. The experimental results revealed that Rest-Net50 outperformed the other pre-trained models and showed a classification accuracy of 96.1% for Dataset-1, 99.5% for Dataset-2, and 99.7% for Dataset-3. Wang et al. [25] used CT images and applied a deep learning algorithm for the clinical diagnosis of the coronavirus disease (COVID-19). They gathered 1065 confirmed COVID-19 CT images and images of other viral pneumonia. They made a necessary modification to the inception transfer-learning model. They also validated their experiments. When the model was tested with internal validation, it showed an accuracy of 89.5%. The specificity and sensitivity were 0.88 and 0.87, respectively. Likewise, external validation achieved 79.3% accuracy with 0.83% specificity and 0.67 sensitivity. Fei Shan et al. [26] proposed a deep learning method that was able to segment the infected regions in chest CT scans, enabling quantitative assessment procedures to make it easier to predict disease severity. This deep-learning-based approach uses VB-Net for the segmentation of the COVID-19 infection region. The model was trained using 246 CT scans of COVID-19 patients. The model was validated by using 300 COVID-19 images. The model was evaluated by calculating performance metrics, such as the Dice similarity coefficient, percentage of infection, and difference in volume. The Dice similarity coefficient of the model was recorded 91.6% ± 10.0%, and the mean POI error estimation was 0.3%. Sethy et al. [27] suggested deep features and a support vector machine for the detection of coronavirus disease (COVID-19) using X-rays. First, the features were extracted using CNN and then fed into the SVM classifier for the classification of COVID-19 patients. This machine learning-based classifier was highly effective for the detection of COVID-19 patients, pneumonia patients, and normal people. The deep features of Res-Net50 yielded superior results for the SVM classifier. The proposed model achieved an accuracy of 95.33%, a sensitivity of 95.33%, and an F1 score of 95.34%. Alakus et al. [28] investigated deep learning using laboratory findings to predict the likelihood of COVID-19 in patients. Data were collected from SARS-CoV-2 patients. The dataset contained data from 5644 patients with 111 laboratory findings. They applied six types of deep learning models: ANN, CNN, LSTM, RNN, CNNRNN, and CNNLSTM. The predictive performance of the model was assessed by calculating the accuracy, F1 score, precision, recall, and AUC. They applied ten-fold cross-validation to the data. Among the six trained deep learning models, LSTM achieved good results and outperformed the other models; AUC score of 62.50 for the prediction of COVID-19 disease. The accuracy of LSTM was 86.66%, the F1-score was 91.89%, and the recall was 99.42%. With the train-test split approach, CNNLSTM showed the highest result, with an accuracy of 92.3%, recall of 93.68%, and recall of 90.00%. Apostolopoulos et al. [29] developed an automatic detection system for COVID-19 using a transfer learning technique. They used X-ray images that contained 224 images of COVID-19 confirmed patients, 504 images of normal persons, and 714 images of patients with confirmed bacterial pneumonia for experimental purposes. They evaluated the performance of a convolutional neural network (CNN) and calculated the accuracy, sensitivity, and specificity of the deep learning model. The model achieved an accuracy of 96.78%, a sensitivity of 98.66%, and a specificity of 96.46%. Amine Amyar et al. [30] proposed a deep-learning-based automatic tool for the classification and segmentation of COVID-19 pneumonia. Three CT image datasets were used to develop the system. This deep learning model could perform multiple tasks, such as the classification of COVID-19 patients and segmentation of COVID-19 lesions from images and reconstruction. The model was validated by comparison with other images. The results achieved by their model were encouraging. The classification accuracy of the model was higher than 94% and the AUC was 0.97. Jain et al. [31] applied deep learning techniques to detect COVID-19 using X-ray images. They developed the models in four phases: data augmentation, preprocessing, stage-1, and stage-2 deep learning design. Initially, they used 1215 images and later augmented the data and increased the number of images to 1832. These deep learning-based models achieved an accuracy of more than 97%. Jain et al. [31] also applied deep learning techniques for the detection of COVID-19 using X-ray images. They developed the models in four phases: data augmentation, preprocessing, stage-1, and stage-2 deep learning design. Initially, they used 1215 images and later augmented the data and increased the number of images to 1832. These deep learning-based models achieved an accuracy of more than 97%. Clinical indicators also play an important role in the prediction of lung diseases. Ali et al. [32] developed a soft voting ensemble-based model to predict exacerbations in patients with lung diseases. They analyzed idiopathic pulmonary fibrosis by applying machine-learning models. They combined three machine learning models: gradient boosting, random forest, and XGBoost. They calculated the accuracy, precision, recall, F1-score, and AUC of the proposed model. This model can predict the severity of IPF in patients with lung cancer. Similarly, Hussain et al. [33] used machine learning techniques to forecast the exacerbation of chronic obstructive pulmonary disease with clinical indicators. Researchers use several other artificial intelligence-based approaches i.e., semantic segmentation, and deep perceptual enhancement for the diagnosis of different kinds of diseases [34,35,36]. Siddhartha et al. [37] developed a method based on white balance and a deep convolutional neural network (DCNN). Contrast limited adaptive histogram equalization (CLAHE) was used to enhance CXR images. They named it COVIDLite. This diagnostic method can be used on mobile devices and is effective for the detection of COVID-19 and pneumonic patients. A total of 1823 images were used in this study; 80% of the dataset was used for training, and 20% was used for testing the model. The architecture of the COVID-Lite architecture consists of two separate convolution blocks. This method can perform both binary and multiclass classifications. For binary classification, it showed a higher accuracy of 99.5%, whereas, for multiclass classification, the accuracy was 96.43%. Because AI-based models are considered black boxes, nobody knows what occurs inside them. If the model predicts something, it does not explain the predictions. Therefore, AI-based models are less commonly used. However, several studies have been conducted to determine the black-box characteristics of artificial intelligence. Explainable artificial intelligence (XAI) has become popular. It can explain how and why a decision has been made that is unanswered in traditional artificial intelligence models. Yang et al. [38] surveyed the current progress of explainable artificial intelligence and proposed solutions through multimodal and multi-center data fusion. The authors also verified the validity of the proposed model. Likewise, to make the deep learning-based model transparent, interpretable, and explainable, N Tsiknakis [39] proposed an AI-based framework for COVID-19 patients using chest X-ray images. For training the Inception V3 model, 115 posteroanterior X-ray images were used. Hyperparameter tuning was performed for the model, which included two main deep learning parts: a convolutional neural network and a standard deep neural network classifier. To enhance the interpretability of the model, the Grad-CAM [40] algorithm was used, which helps visualize, examine, and analyze the importance of each pixel. This pre-trained model achieved a 100% AUC for binary classification while detecting patients with COVID-19. Muhammet Fatih Aslan et al. [41] applied CNN and Bayesian optimization for the diagnosis of COVID-19 using X-ray images. They extracted the features from the images using different CNN-based models for example AlexNet, ResNet Inceptionv3, GoogleNet, etc. They also applied data augmentation for data diversity. They used four ML algorithms for classification purposes. Their SVM model achieved the highest accuracy of 96.29%. Shamik Tiwari et al. [42] proposed deep learning for the detection of COVID-19 from CT scans of the lungs. They performed COVID-19 detection by combining a capsule network with different types of CNN e.g., ResNet, VGGNet, DenseNet, and MobileNet. Their proposed models achieved 99%. Table 1 shows the comparison of some related works for the detection of COVID-19.

## 3. Methods and Materials

In this section, we elucidate the materials and methods utilized in this research work. We developed an automated system for the prediction of COVID-19. This system has four main steps. First, we extracted the region of interest (ROI) of the images. Second, we applied a biased field estimation and correction algorithm to correct the intensities of the X-ray images. Third, we developed the DCSCNN model for differentiating COVID-19 patients from pneumonia, lung opacity, and normal. Finally, we used Grad-Cam and Lime techniques for the interpretation of our proposed model.

### 3.1. Data Source and Description

**Figure 1:**
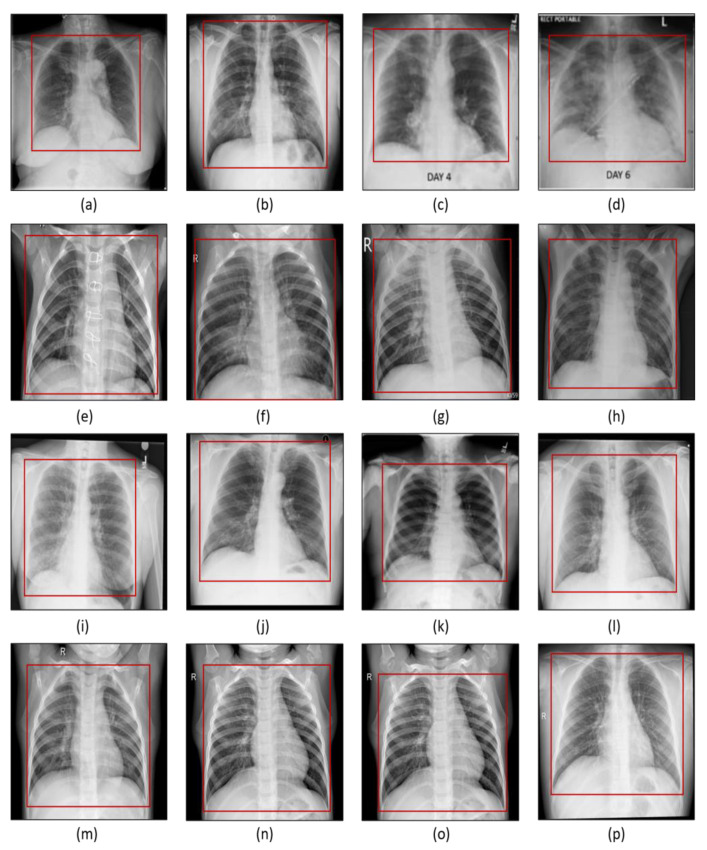
(**a**–**d**) COVID-19 chest X-ray, (**e**–**h**) pneumonia chest X-ray, (**i**–**l**) lung opacity chest X-ray, (**m**–**p**) normal chest X-ray. The red box signifies the region of interest cropped from the original image for analysis.

The data source and the details about the data are explained in this section. Four datasets were used which are discussed here. From each dataset, we chose 2500 images that were used in our research study. Figure 1 shows the examples of samples of each dataset used for this research work.

#### 3.1.1. COVID-19 Dataset

A database was developed by a team of researchers from different universities in different countries such as Qatar University, UET Peshawar Pakistan, University Kebangsaan Malaysia, and Dhaka University Bangladesh. The COVID-19 dataset was created by gathering CXR images of COVID-19 positive patients from different sources such as published articles, online sources, and other publicly available datasets. This dataset consisted of 3616 images. A total of 2473 images were obtained from the BIMCV-COVID19+ dataset [50]. 560 X-ray images were collected from the Italian Society of Medical Radiology, Kaggle, Github, and Twitter [51]. A total of 183 and 400 images were obtained from a German medical school and another COVID-19 CXR repository, respectively.

#### 3.1.2. Normal Dataset

The normal (healthy) person dataset consisted of 10,192 images which were gathered from three different data sources. Of these 10,192 images, 8851 were taken from the Radiological Society of North America RSNA [52] whereas 1341 were collected from Kaggle [53].

#### 3.1.3. Lung Opacity Dataset

There were 6012 images of lung opacity collected from the Radiological Society of North America (RSNA) [52] CXR dataset.

#### 3.1.4. Pneumonia Dataset

This dataset was collected from the Kaggle chest X-ray database which contained 3906 patients affected by pneumonia.

### 3.2. Preprocessing

Preprocessing is one of the most important steps in the development of ML or DL models. The model produces optimal performance when trained on high-quality data. Low-quality data may cause model overfitting or underfitting issues. Preprocessing has other benefits, such as reducing the model training time and increasing the inference speed of the model. X-ray images that are taken from different databases, or sources may have different formats or qualities. Therefore, they must be preprocessed and converted to good quality and format before using them for any predictive or diagnostic purpose. Medical image enhancement helps experts and professionals to make precise and accurate decisions regarding the diagnosis and prognosis of the disease. Thus, there is a need to improve the quality of the image.

In image preprocessing, the aim is to enhance the diminishing features of the image using different image processing techniques thus improving the quality of the image to be used for training the model. All the X-ray images used in this research study were cropped (Figure 1) and resized to extract the ROIs and have identical widths and heights before being fed to the learning algorithm. Moreover, a bias field correction technique is applied to correct the inhomogeneous intensity in the image.

#### 3.2.1. Bias Field Correction

**Figure 2:**
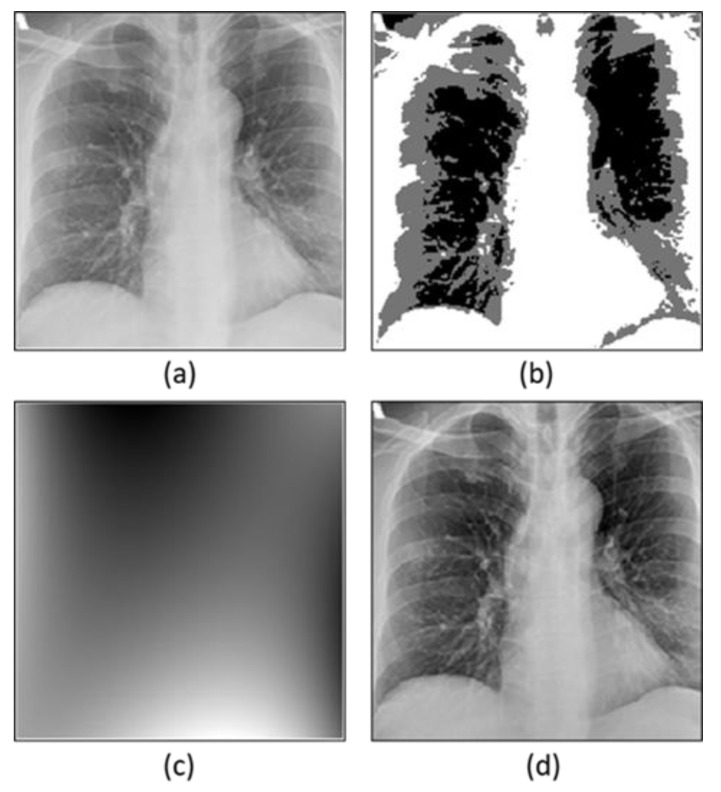
Application of bias field correction of the X-ray image. (**a**) Original image, (**b**) segmentation of inhomogeneous intensity, (**c**) estimated bias field image, (**d**) bias field correction image.

Bias field correction is an approach used to enhance the quality of an image [54]. Mostly there are intensity in-homogeneity issues in the images because of the bias field signals. These bias field signals are the signals with low frequency, and they are responsible for bringing down the high-frequency information thus reducing the quality of images. Therefore, bias field correction is used to address this problem which is based on energy minimization operation. The bias field correction is applied in two steps by decomposing the images into two multiplicative components. First, the estimation of the bias field is performed and second, bias field correction is conducted (Figure 2). The energy minimization strategy [55,56] is used to optimize these two components.

##### Optimization of Multiplicative Intrinsic Componen

Optimization of multiplicative intrinsic component is generally considered an image I that can be expressed as:

(1) I(x)=b(x)J(x)+n(x)

where *I*(*x*) denotes the observed image intensity at voxel x. The bias field is represented by *b*(*x*) and it is responsible for the intensity inhomogeneity in the observed image. *J*(*x*) represents the true image and *n*(*x*) is the noise or additive distortion with zero mean. It is assumed that the bias field *b* varies smoothly. The components *b* and *j* are considered as the two intrinsic multiplicative components of the observed image *I*. Bias field estimation is performed by calculating the linear combination of optimal coefficients *w* = *w*_1_, …, *w_M_* and smooth basis functions *G*(*x*) = *g_1_*, …, *g_M_* using a column vector *w*. Here *T* represents the transpose. The bias field *b*(*x*) can be formulated as:

(2) b(x)=w^T G(x)

Furthermore, the true image *J* can be expressed by $J\left(x\right)=\overset{N}{\underset{i=1}{\sum }}c_{i}u_{i}\left(x\right)$ representing membership function as *u_i_* and constant as *c_i_*. The bias field corrected image is generated using the estimated bias field *b*. The energy minimization *F*(*b*,*J*) is expressed as:

(3) $$F\left(b,\;J\right)=F\left(u,c,\;w\right)=\underset{\Omega }{\int }{\left|I\left(x\right)-w^{T}G\left(x\right)\overset{N}{\underset{i=1}{\sum }}c_{i}u_{i}\left(x\right)\right|}^{2}dx$$

The optimization of *b* and *j* can be achieved by minimizing the energy *F* with respect to the variables *u*, *c*, and *w*.

##### Minimization of Energy

Energy minimization can be obtained by minimizing the energy function *F*(*u*,*c*,*w*) with respect to one of its variables while keeping the other two variables constant.

In this scenario variables, *u* and *w* are being fixed with respect to *c* for the energy *F*(*u*,*c*,*w*).

(4) $${\hat{c}}_{i}=\frac{\int_{\Omega }I\left(x\right)b\left(x\right)u_{i}\left(x\right)d\left(x\right)}{\int_{\Omega }b^{2}\left(x\right)u_{i}\left(x\right)d\left(x\right)},\;i=1,\ldots N$$

Optimization of *w* can be achieved by fixing the variables *c* and *u*,

(5) $$\hat{w}=\underset{\Omega }{\int }G\left(x\right)G^{T}\left(x\right){\left(\overset{N}{\underset{i=1}{\sum }}c^{2i}u^{2i}\left(x\right)\right)}^{-1}d\left(x\right)\underset{\Omega }{\int }.G\left(x\right)I\left(x\right)\overset{N}{\underset{i=1}{\sum }}c^{2i}u^{2i}\left(x\right)\;d\left(x\right)\;$$

The following equation is used for the prediction of the bias field,

(6) $$\hat{b}\left(x\right)={\hat{w}}^{T}G\left(x\right)\;$$

Optimization of *u* can be achieved while fixing the variable *w* and *c* of energy minimization expression *F*,

(7) $${\hat{u}}_{i}=\frac{{\left(\delta_{i}\left(x\right)\right)}^{\frac{1}{1-q}}}{\sum_{j=1}^{N}{\left(\delta_{i}\left(x\right)\right)}^{\frac{1}{1-q}}}\;i=1,\ldots ,N$$

where $\delta_{i}\left(x\right)={\left|I\left(x\right)-w^{T}G\left(x\right)c_{i}\right|}^{2}$

### 3.3. Convolutional Neural Network

**Figure 3:**
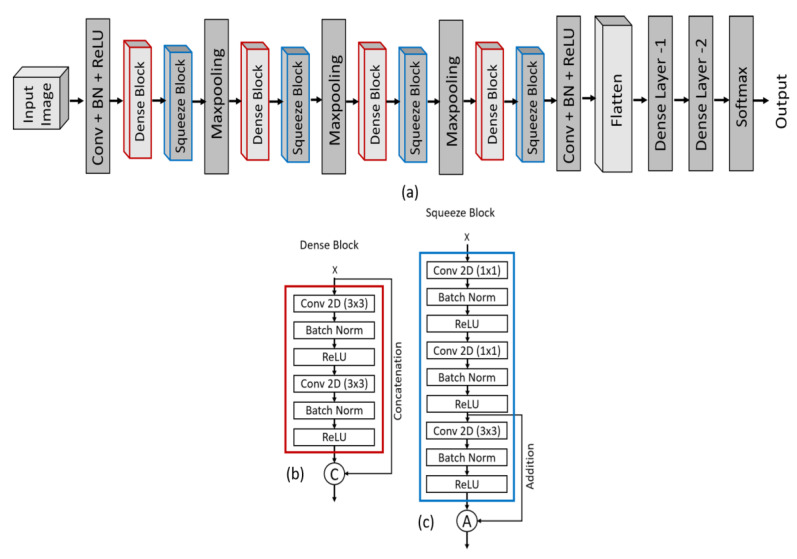
The entire architecture of the proposed DCSCNN model. (**a**) Design of convolution neural network with two fully connected layers. (**b**) Dense block with concatenation function. (**c**) Squeeze block with additional function. In dense block, the term “x” is the input layer of Convolution, Batch normalization, and ReLU, while in squeeze block, the term “x” is the output of the dense block.

**Figure 4:**
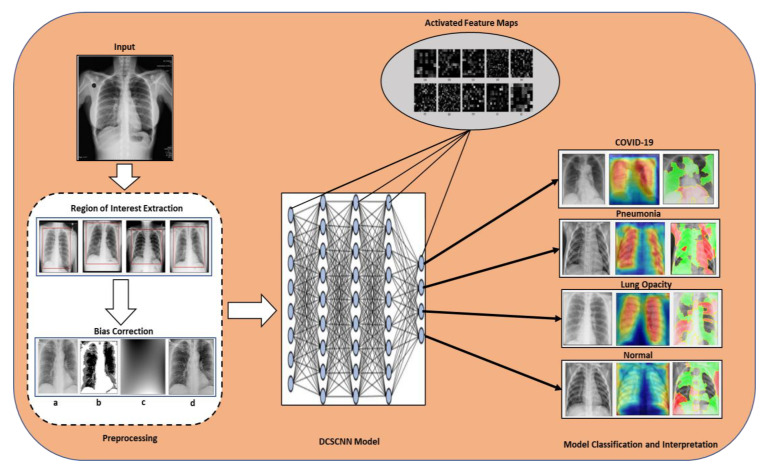
The overall architecture of the proposed densely connected squeezed convolutional neural network (DCSCNN) model.

In recent years, deep learning has been widely used for the classification of medical images for the diagnosis and prediction of different types of diseases. The diagnosis and prediction accuracy of DL models are very high, fast, and accurate therefore, they are gaining popularity in the field of healthcare as well. Considering the importance of deep learning, we propose a CNN-based model in this research study for the classification of COVID-19 patients. To construct the proposed DL model, we used a CNN with TensorFlow-GPU and Keras library [57]. Our proposed model consists of 22 convolutional layers with a rectified linear unit (ReLU) activation function, 22 batch normalization layers, four concatenation layers, four additional layers, three max-pooling layers, a flattened layer, two dense layers, and a classification layer with a softmax activation function for binary and multiclass classification. This architecture consists of four dense blocks and squeeze blocks with different numbers of convolutional layers. Model learning was performed with the Adam optimizer [58] and eight batch sizes for 100 epochs, and after each epoch, the model was automatically saved based on the lowest validation loss which was later used for testing. The convolutional layer comprises different numbers of filters of a certain size. These filters move across the whole image with a certain stride and extract the high-level features from the image. The pooling layer helps to extract only the dominating features thus reducing the computational power and enhancing the performance of the model. The flattening layer is used to convert the 2D array into a 1D array and the dense layer of CNN is the fully connected layer that compiles the data extracted by previous layers to form the final output. Dense block and squeeze block modules have been utilized in our model to improve the representational power of the network, improve the vanishing gradient problem, and strengthen the feature propagation. The idea of dense and squeeze blocks has been taken from the DenseNet [59] and SqeezeNet [60] architecture. DenseNet is a kind of convolutional neural network that uses dense connections between different layers in a feed-forward fashion. It has several advantages, for example, reduces the vanishing gradient problem and also reduces the number of parameters. Furthermore, it encourages the reuse of features. Likewise, SqeezeNet is also a type of CNN but has smaller architecture and a good level of accuracy. It has more efficient distributed training, less overhead, and feasible FPGA and embedded deployment. The specification and internal design of our DCSCNN architecture are shown in Table 2 and Figure 3, respectively. Furthermore, the overall architecture of our proposed model has been shown in Figure 4.

## 4. Experimental Results and Discussion

All the experiments of our research study were conducted on a personal computer Intel(R) Core (TM) i7-7700 CPU @ 3.60 GHz processor with 64-bit windows10 operating system, 32 GB RAM, and NVIDIA GeForce RTX 3060 graphics processing unit card. We have used MATLAB (ver. R2020b; MathWorks, Natick, MA, USA) for the data pre-processing (i.e., inhomogeneity correction), and Python (ver. 3.8.6) for data augmentation and implementation of the deep learning model using Scikit learn, Keras, TensorFlow libraries. The classification performance of the proposed model was evaluated using accuracy, precision, recall, F1-score, Cohen’s kappa (K), and receiver operating characteristic (ROC) curve.

**Figure 5:**
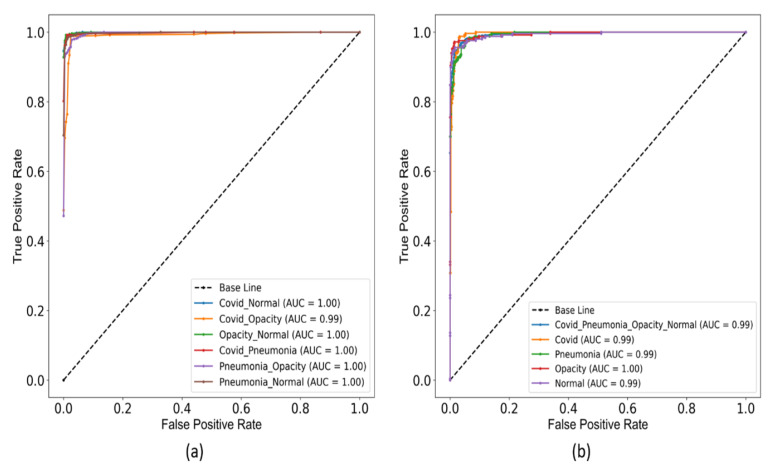
Receiver operating characteristic (ROC) curves on the test dataset. (**a**) Binary classification. (**b**) Multiclass classification.

A total of 19,820 X-ray images consisting of COVID-19, pneumonia, lung opacity, and normal patients were collected from different sources. Out of these, 10,000 (i.e., 2500 for each class) images were selected manually to balance the dataset. Further, to train and test the CNN model, we split the binary and multiclass datasets according to a 9:1 ratio. Table 3 shows the scores of binary and multiclass classifications of the test data set. From the performance analysis, it was found that the model showed the best result in classifying COVID-19 and normal patients and achieved an overall accuracy, precision, recall, f1-score, and K of 98.8%, 98.8%, 98.8%, 98.8%, and 97.6%, respectively. In Supplementary Information (SI), Tables S1–S7 shows the confusion matrices generated based upon the results of each binary and multiclass classification, separately. Figure 5a,b shows the ROC curve of binary and multiclass classification, respectively.

Generally, precision and recall measures are considered to analyze the performance of the prediction models. Therefore, from the confusion matrices, as shown in SI, Tables S1–S3 and S7, it can be observed that the precision of the samples belonging to the COVID-19 patient class is higher in all the cases (i.e., COVID-19 vs. normal, COVID-19 vs. lung opacity, COVID-19 vs. pneumonia, and COVID-19 vs. pneumonia vs. lung opacity vs. normal) which means our proposed model performed well in identification of COVID-19 samples from chest X-ray images. Moreover, we also used the ROC curve to evaluate the performance of the proposed CNN model. In SI, Figure S1 shows the ROC curve of the prediction model of each binary classification, separately.

**Figure 6:**
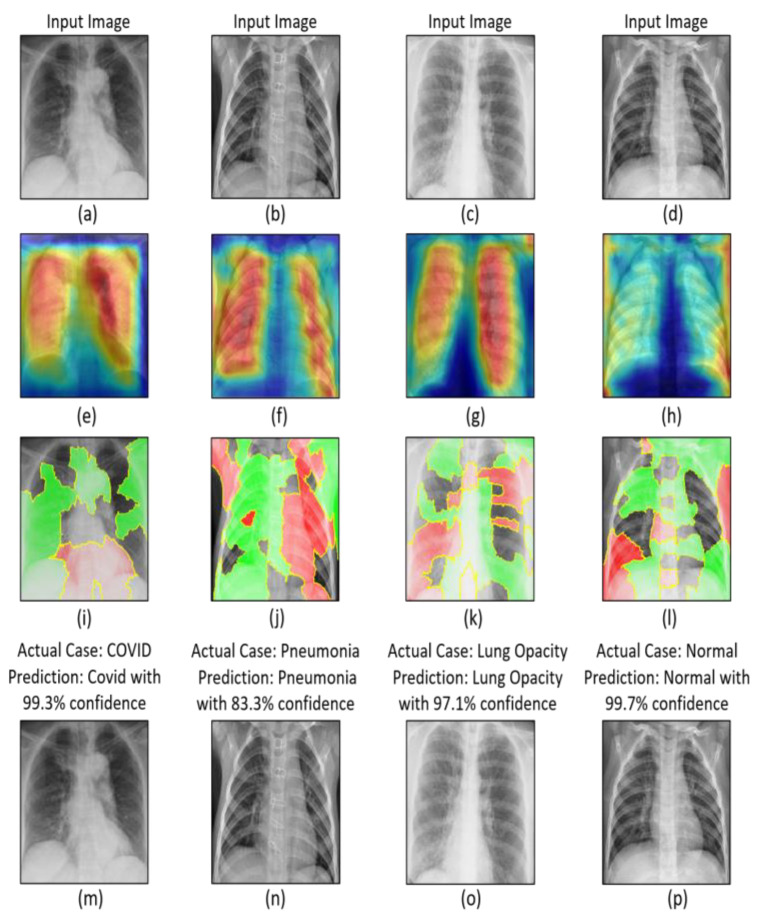
Visualization of significant regions of CNN. (**a**) COVID-19. (**b**) Pneumonia. (**c**) Lung opacity. (**d**) Normal (**e**–**h**) The results of Grad-CAM method for (**a**), (**b**), (**c**), and (**d**), respectively. (**i**–**l**) The results of LIME method for (**a**), (**b**), (**c**), and (**d**), respectively. (**m**–**p**) The predicted samples of (**a**), (**b**), (**c**), and (**d**), respectively.

In the field of computer vision, visualization results are essential for understanding the model’s prediction. The interpretable and explainable techniques used in this study to understand the internal behavior of a CNN model are Grad-CAM and LIME. Nonetheless, the visualizing results give us good intuition about neural networks and show the attention regions where the model focuses on the role of significant features. In SI, Figure S2 shows the visualization results of different activation layers in the CNN model, and it can be observed which feature maps are getting activated in each layer in the model. In addition, Grad-CAM [61] and LIME [62] techniques were adopted to visualize the attention regions and investigate the contribution of super-pixels for the prediction, as shown in Figure 6. Therefore, we can observe that interpretable and explainable techniques tended to pay more attention to the discriminative part of the images.

In this research work, we presented a deep learning-based densely connect squeeze convolutional neural network (DCSCNN) for the detection of COVID-19 patients. Four kinds of data (i.e., COVID-19, lung opacity, pneumonia, and normal) were collected from different sources. Although the source databases have a huge amount of data, however, we chose only 10,000 X-ray images of good quality so that we can gain good performance of our model.

For each class, we selected 2500 images. The data were further preprocessed using different preprocessing techniques. The bias field correction technique was used to enhance the quality of images. To maintain the transparency, interpretability, and explainability of the model, we used the Grad-CAM and LIME methods. These approaches provide visualization of the attention regions and decisions of the AI model. Unlike the traditional ML or DL models, the proposed model is capable of interpreting and justifying the decisions it makes and provides clear and transparent predictions. Our proposed DCSCNN combines the strength of the dense and squeeze networks which help to improve the performance and validity of the model. The model performed six binary classifications and one multiclassification of COVID-19, lung opacity, pneumonic, and normal patients. The model was trained several times by performing hyperparameter tuning of the model and choosing the best-performing hyperparameters. Different performance measure metrics such as accuracy, precision, recall, F1-score, and Kappa coefficient were calculated for each classification. The overall classification accuracy for COVID-19 vs. normal, COVID-19 vs. pneumonia, COVID-19 vs. lung opacity, lung opacity vs. normal, pneumonia vs. lung opacity, pneumonia vs. normal, and COVID-19 vs. pneumonia vs. lung opacity vs. normal are 98.8%, 96.4%, 98.2%, 97.2%, 95.8%, 97.4%, and 94.7%, respectively.

## 5. Conclusions

In the wake of this widespread COVID-19 pandemic, an accurate, fast, and reliable automatic system is urgently needed to contain the spread of this dreadful disease and for its early diagnosis. This research work investigated a densely connected squeeze convolutional neural network (DCSCNN) for the prediction of COVID-19 patients using X-ray images collected from different databases. This model could perform seven types of classifications in which six are binary classifications (COVID vs. normal, COVID vs. lung opacity, lung opacity vs. normal, COVID vs. pneumonia, pneumonia vs. lung opacity, pneumonia vs. normal) and one was a multiclass classification (COVID vs. pneumonia vs. lung opacity vs. normal). To enhance the quality of the images and obtain better results from the model, we used bias field correction, and other data preprocessing techniques. Grad-CAM and LIME techniques were used to maintain the transparency and explainability of the model. These methods helped us visualize the attention regions and investigate the contribution of super pixels to the prediction.

Among all the seven classifications, COVID-19 vs. normal achieved the highest accuracy with 98.8% followed by COVID-19 vs. lung opacity with 98.2%, lung opacity vs. normal with 97.2%, COVID-19 vs. pneumonia with 96.4%, pneumonia vs. lung opacity with 95.8%, pneumonia vs. normal with 97.4%, and COVID vs. pneumonia vs. lung opacity vs. normal with 94.7%. Moreover, the precision, recall, f1-score, and kappa coefficients, and AUC were also calculated, and the performance of the model was evaluated. This proposed model will mitigate the COVID-19 pandemic situation. Unlike the traditional diagnosis system i.e., RT-PCR, our model is fast, accurate, and efficient. It also resolves the problem of the unavailability of RT-PCR kit. Furthermore, it will provide decision-making assistance to physicians and doctors with high performance and will deliver quick and proactive solutions for COVID-19 patients and other lung disease patients.

## Figures and Tables

**Table 1:** Comparison of some other related works.

Authors	Technique Used	Accuracy
L Wang et al. [43]	COVID-NET	93.3%
Y Oh et al. [44]	Patch-based CNN	93.3%
X Li et al. [45]	COVID-MobileXpert	93.5%
MA Alves et al. [46]	Decision Tree explainer, RF	88%
A Banerjee et al. [47]	RF, LR, GLMNET, ANN	81–87%
A Abbas et al. [48]	Decompose, Transfer, and Compos	95.12%
VA de Freitas Barbosa et al. [49]	XMLP, SVM, RT, RF, BN, NB	95.159%

**Table 2:** Specification of DCSCNN Architecture.

Layers	Output Size	DCSCNN
Input	256 × 256	-
Convolution (1)	128 × 128	5 × 5 conv, stride 2, filter 64
Dense Block (1)	128 × 128	[3 × 3 conv] × 2, filter 128, 128
Squeeze Block (1)	128 × 128	$\left[\begin{array}{c} 1\times 1\;conv\\ 1\times 1\;conv\\ 3\times 3\;conv \end{array}\right]$, filter 64, 128, 128
Max Pooling (1)	128 × 128	2 × 2, stride 2
Dense Block (2)	64 × 64	[3 × 3 conv] × 2, filter 256, 512
Squeeze Block (2)	64 × 64	$\left[\begin{array}{c} 1\times 1\;conv\\ 1\times 1\;conv\\ 3\times 3\;conv \end{array}\right]$, filter 128, 256, 256
Max Pooling (2)	32 × 32	2 × 2, stride 2
Dense Block (3)	32 × 32	[3 × 3 conv] × 2, filter 512, 256
Squeeze Block (3)	32 × 32	$\left[\begin{array}{c} 1\times 1\;conv\\ 1\times 1\;conv\\ 3\times 3\;conv \end{array}\right]$, filter 256, 512, 512
Max Pooling (3)	16 × 16	2 × 2, stride 2
Dense Block (4)	16 × 16	[3 × 3 conv] × 2, filter 128, 128
Squeeze Block (4)	16 × 16	$\left[\begin{array}{c} 1\times 1\;conv\\ 1\times 1\;conv\\ 3\times 3\;conv \end{array}\right]$, filter 64, 128, 128
Convolution (2)	16 × 16	1 × 1 conv, filter 64
Classification Block	16 × 16	FC (32)
		FC (16)
		FC (2 & 4), SoftMax

**Table 3:** Performance analysis of DCSCNN Model between binary and multiclass classification.

Dataset	Accuracy (%)	Precision (%)	Recall (%)	F1-Score	K (%)
COVID-19 vs. Normal	98.8	98.8	98.8	98.8	97.6
COVID-19 vs. Lung Opacity	98.2	98.2	98.3	98.3	96.4
Normal vs. Lung Opacity	97.2	97.2	97.3	97.3	94.4
COVID-19 vs. Pneumonia	96.4	96.4	96.6	96.5	92.8
Lung Opacity vs. Pneumonia	95.8	95.8	96.1	95.9	91.6
Normal vs. Pneumonia	97.4	97.4	97.5	97.4	94.8
COVID-19 vs. Pneumonia vs. Lung Opacity vs.Normal	94.7	94.7	94.8	94.8	92.8

K = Cohen’s kappa.

## Data Availability

The data presented in this study are available in the article and Supplementary Materials.

## Supplementary Materials

The following supporting information can be downloaded at: https://www.mdpi.com/article/10.3390/s22249983/s1.
