# Supplementary material for: Detection of COVID-19 in X-ray Images Using Densely Connected Squeeze Convolutional Neural Network (DCSCNN): Focusing on Interpretability and Explainability of the Black Box Model
Source: Sensors (Basel). 2022 Dec 18;22(24):9983. doi: 10.3390/s22249983 (PMC9781899; doi:10.3390/s22249983)
Supplement: Supplementary file 1 [file sensors-22-09983-s001.zip › sensors-2058457-supplementary.pdf]

Supplementary Material

Supplementary Information (SI)

Appendix

Figures

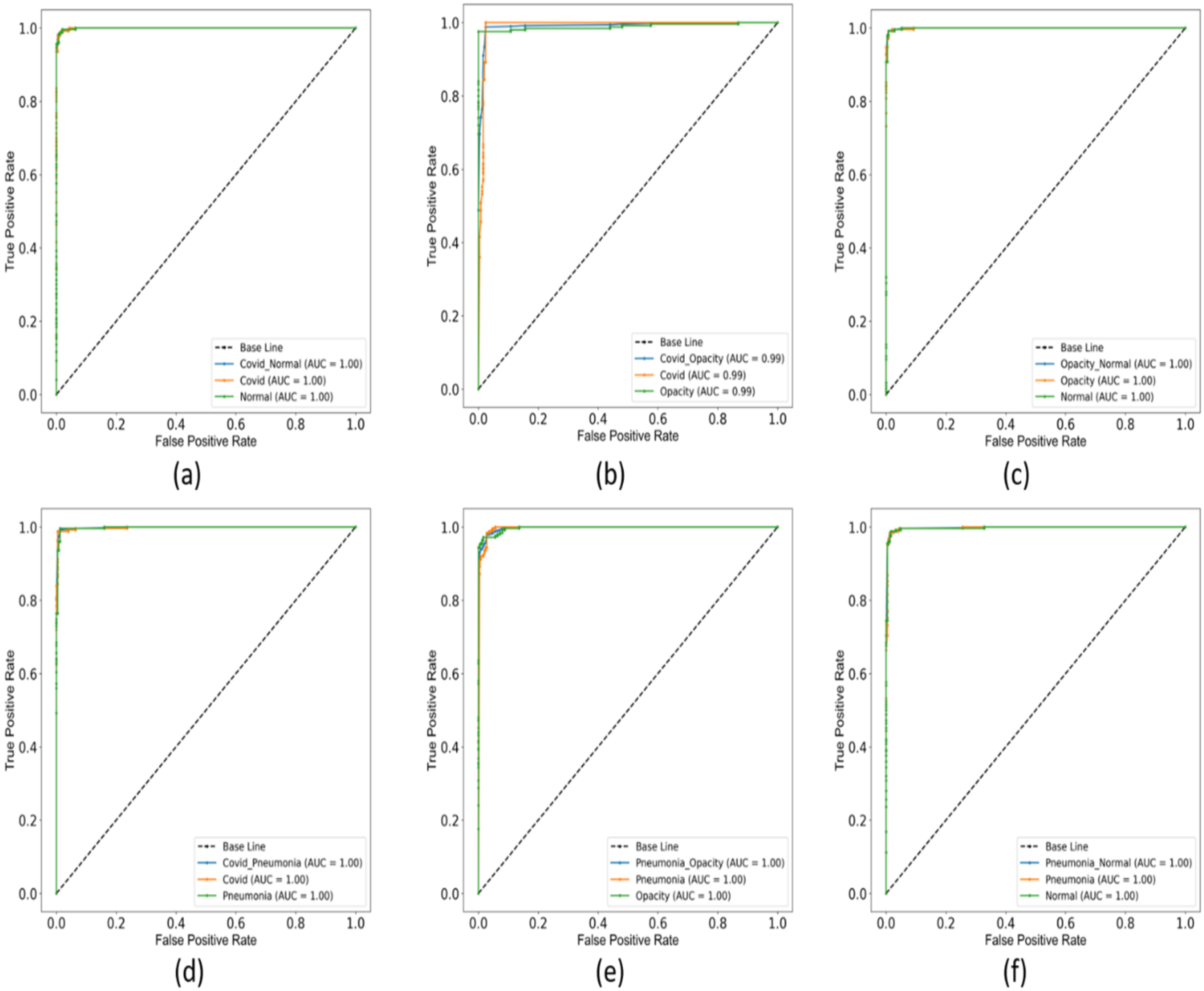

Figure S1. Receiver operating characteristic (ROC) curves of binary classification on test dataset.

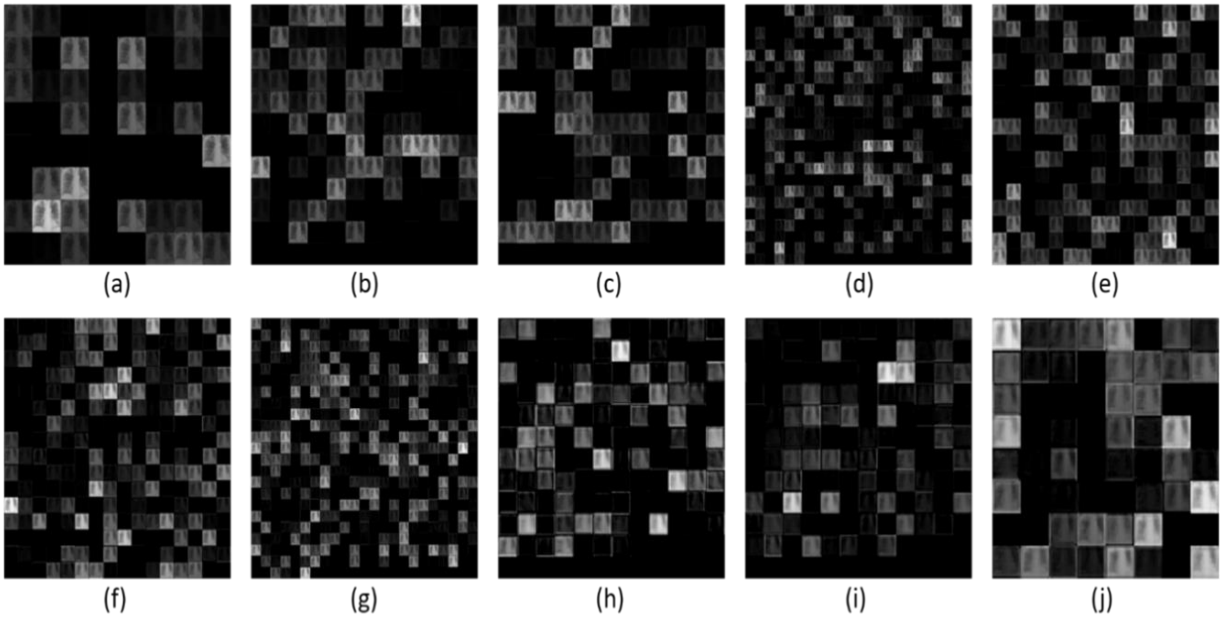

Figure S2. Activated feature maps generated from the different activation layers of the CNN model. (a) Activation layer 1. (b) Activation layer 2. (c) Activation layer 5. (d) Activation layer 7. (e) Activation layer 10. (f) Activation layer 12. (g) Activation layer 15. (h) Activation layer 17. (i) Activation layer 20. (j) Activation layer 21.

Table S1

Confusion Matrix of DCSCNN Model for Covid-19 vs. Normal.

Binary Class		Predicted		
		COVID-19	Normal	Overall Classification
Actual	COVID-19	248	2	250
	Normal	4	246	250

Table S2

Confusion Matrix of DCSCNN Model for Covid-19 vs. Lung Opacity.

Binary Class		Predicted		
		COVID-19	Lung Opacity	Overall Classification
Actual	COVID-19	250	0	250
	Lung Opacity	9	241	250

Table S3

Confusion Matrix of DCSCNN Model for Lung Opacity vs. Normal.

Binary Class		Predicted		
		Lung Opacity	Normal	Overall Classification
Actual	Lung Opacity	249	1	250
	Normal	13	237	250

Table S4

Confusion Matrix of DCSCNN Model for Covid-19 vs. Pneumonia.

Binary Class		Predicted		
		COVID-19	Pneumonia	Overall Classification
Actual	COVID-19	249	1	250
	Pneumonia	17	233	250

Table S5

Confusion Matrix of DCSCNN Model for Pneumonia vs. Normal.

Binary Class		Predicted		
		Pneumonia	Normal	Overall Classification
Actual	Pneumonia	249	1	250
	Normal	12	238	250

Table S6

Confusion Matrix of DCSCNN Model for Pneumonia vs. Lung Opacity.

Binary Class		Predicted		
		Pneumonia	Lung Opacity	Overall Classification
Actual	Pneumonia	250	0	250
	Lung Opacity	21	229	250

Table S7

Confusion Matrix of DCSCNN Model for Covid-19 vs. Pneumonia vs. Lung Opacity vs. Normal.

Multiclass		Predicted				
		COVID-19	Pneumonia	Lung Opacity	Normal	Overall Classification
Actual	COVID-19	243	7	0	0	250
	Pneumonia	14	231	0	5	250
	Lung Opacity	4	8	238	0	250

	Normal	4	4	7	235	250
